# Explainable machine learning for preoperative prediction of acute kidney injury after intertrochanteric fracture surgery in older adults

**DOI:** 10.3389/fmed.2026.1961926

**Published:** 2026-10-09

**Authors:** Weizhang Lin, Ximing Lin, Hongpeng Gao

**Affiliations:** 1 Department of Orthopaedic Surgery, Fujian Medical University Affiliated Zhangzhou Hospital, Zhangzhou, Fujian, China; 2 The Second Clinical Medical College, Fujian Medical University, Zhangzhou, Fujian, China

**Keywords:** acute kidney injury, explainable artificial intelligence, intertrochanteric femoral fracture, machine learning, model calibration, research prototype, risk prediction, SHAP

## Abstract

**Background:**

Acute kidney injury (AKI) is an important postoperative complication in older patients undergoing surgery for intertrochanteric femoral fracture. This study aimed to develop and internally evaluate an explainable model for preoperative AKI risk assessment.

**Methods:**

This retrospective study included 616 patients aged ≥60 years, including 54 (8.8%) who developed postoperative AKI. Candidate predictors were processed and selected using development-cohort data, and seven machine-learning algorithms were compared. Model performance was assessed across 100 repeated stratified 70/30 partitions and by 500-repetition model-specific and complete-pipeline bootstrap analyses. Calibration, decision-curve analysis, and SHAP were used to evaluate and interpret the selected model.

**Results:**

The reported Naive Bayes model comprised baseline serum creatinine, blood urea nitrogen, chloride, uric acid, and chronic kidney disease or renal insufficiency. Across 100 repeated partitions, its mean validation AUC was 0.7231 (SD, 0.0260). The optimism-corrected AUC was 0.8004 for the fixed five-predictor Naive Bayes specification and 0.756 when the complete development pipeline was repeated; the latter analysis yielded a corrected Brier score of 0.084, calibration intercept of 0.02, and calibration slope of 1.70. In the original algorithm-comparison cohort, the observed AUC was 0.795 (95% CI, 0.698–0.892). When the first serum creatinine measurement obtained after admission was used as the alternative reference value, the number of AKI events decreased from 54 to 43 and the validation AUC was 0.706 (95% CI, 0.586–0.826).

**Conclusion:**

The model showed moderate internal discrimination across repeated partitions. Model-specific and complete-pipeline bootstrap analyses provided complementary estimates of internal optimism. The model may complement routine preoperative renal assessment while its calibration and transportability are evaluated in additional cohorts.

## 1 Introduction

Hip fracture is a major consequence of skeletal fragility in later life and is associated with functional loss, substantial healthcare use, and excess mortality. Intertrochanteric fractures predominantly affect older adults with frailty, multimorbidity, and limited physiological reserve (1, 2). Although surgical repair enables stabilization and mobilization, the perioperative period combines trauma, fasting, fluid shifts, blood loss, anesthesia, and inflammation. These concurrent stresses can compromise renal perfusion and metabolic homeostasis.

Postoperative AKI is a clinically important complication after hip fracture surgery. Reported incidence and associated factors vary with cohort age, fracture type, outcome definition, and perioperative assessment. Prior studies have identified baseline renal dysfunction, CKD, BUN, cardiovascular comorbidity, and anemia as relevant predictors (3–6). Transfusion, overall comorbidity burden, and intraoperative factors have also been implicated (7, 8), and recent models have combined several of these characteristics (9, 10). AKI is associated with longer hospitalization and higher short- and long-term mortality (11). Hemodynamic instability provides an additional context for perioperative renal vulnerability (12). These observations support a focused evaluation of preoperative prediction in older adults undergoing intertrochanteric fracture surgery.

AKI is a heterogeneous syndrome arising from interactions among renal reserve, perfusion, inflammation, tubular stress, and exposure to nephrotoxic or hemodynamically active treatments. It is associated with subsequent CKD and adverse outcomes (13, 14), while clinical ascertainment relies mainly on changes in serum creatinine and urine output (15). These interacting processes are unlikely to be captured by a single preoperative measurement.

Machine-learning methods may capture nonlinear and interacting clinical patterns that are difficult to represent with a single conventional specification. Electronic-health-record models have been developed for AKI prediction in hospitalized populations (16, 17). A commentary has outlined opportunities and methodological challenges in machine-learning-based AKI prediction (18). An explainable preoperative model has also been reported for cardiac surgery-associated AKI (19). Recent work has integrated feature selection, model comparison, calibration, and explainability within a single prediction workflow (20). Transparent reporting, calibration, decision analysis, and separation of prediction from causation remain essential (21). We therefore developed and internally evaluated an explainable preoperative model for postoperative AKI after intertrochanteric fracture surgery and translated the fitted workflow into an R Shiny research prototype.

## 2 Materials and methods

### 2.1 Study design and participants

This single-center retrospective cohort study included patients aged 60 years or older who underwent surgery for an intertrochanteric femoral fracture at Fujian Medical University Affiliated Zhangzhou Hospital between January 2015 and December 2025. Fractures were confirmed from clinical and radiographic records. Eligibility required a preoperative serum creatinine measurement and at least one serum creatinine measurement within 7 days after surgery. Exclusion criteria were pathological or periprosthetic fracture, AKI before surgery, maintenance dialysis, previous kidney transplantation, or insufficient renal data for outcome ascertainment. The institutional ethics committee approved the study and waived individual informed consent because existing clinical records were analyzed retrospectively. Figure 1 summarizes cohort assembly and the analytical workflow.

**Figure 1:**
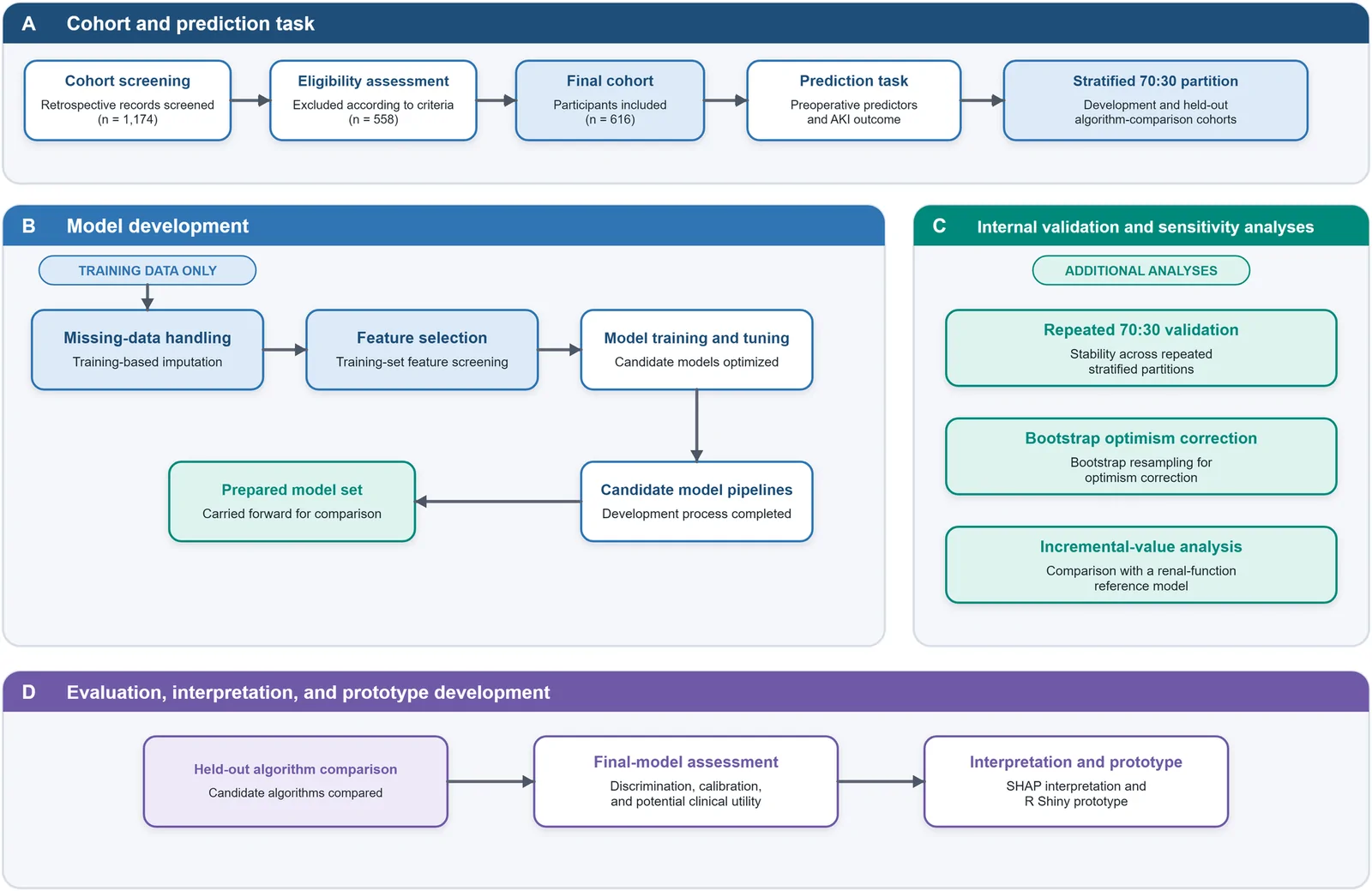
Study workflow. (**A**) Cohort selection, eligibility assessment, prediction task, and data partitioning. (**B**) Model development using development-cohort data. (**C**) Internal validation and sensitivity analyses. (**D**) Held-out algorithm comparison, final-model assessment, interpretation, and prototype development. Imputation, feature selection, tuning, and model fitting used only development-cohort information.

### 2.2 Outcome and candidate predictors

Postoperative AKI was defined using the serum-creatinine component of the 2012 Kidney Disease: Improving Global Outcomes (KDIGO) criteria (15) as an increase in serum creatinine of at least 0.3 mg/dL (26.5 μmol/L) within 48 h or an increase to at least 1.5 times the baseline value within 7 days. Baseline serum creatinine was defined as the lowest measurement obtained between hospital admission and surgery. This reference was chosen because admission creatinine may be transiently elevated by dehydration, acute physiological stress, or other potentially reversible prerenal factors. It represented the lowest observed preoperative serum creatinine concentration after admission and initial management, rather than admission renal function or a confirmed premorbid baseline. This approach was informed by the pragmatic guidance in KDIGO Chapter 2.4 when a reliable premorbid creatinine value is unavailable. KDIGO also notes that hemodilution after substantial fluid resuscitation may lower serum creatinine and complicate its interpretation. Urine-output criteria were not applied because sufficiently detailed and consistently recorded urine-output data were unavailable. Postoperative serum creatinine measurements were obtained as part of routine clinical care. When multiple measurements were available, all values within the relevant KDIGO time windows were evaluated against the baseline value.

Candidate predictors included age, sex, body mass index, preoperative waiting time, anesthesia type, comorbidities, and routine preoperative hematological, biochemical, electrolyte, liver-function, and coagulation measurements. Laboratory predictors other than baseline creatinine were represented by the single preoperative values recorded in the analytic dataset. The final model was intended for use after these preoperative results were available and before surgery. Comorbidities included hypertension, diabetes mellitus, coronary artery disease or myocardial infarction, cardiac insufficiency or heart failure, and CKD or renal insufficiency. CKD or renal insufficiency was defined from the admission medical record or preoperative medical history rather than from a single perioperative serum creatinine or estimated glomerular filtration rate measurement. Operative duration was evaluated among the 41 candidate variables and entered the imputation and Boruta screening workflow.

### 2.3 Missing data and preprocessing

Baseline tables used observed, unimputed values. For prediction modeling, multiple imputation by chained equations generated five completed datasets (22). Predictive mean matching was used for continuous variables, logistic regression for binary variables, and polytomous regression for multilevel categorical variables. Imputation and preprocessing relied only on development-cohort information, and the outcome was not used as an imputation predictor. Categorical predictors were dummy encoded, while continuous predictors were centered and scaled using development-set parameters. The analysis assumed that data were missing at random conditional on the observed demographic, clinical, and laboratory information.

### 2.4 Feature selection and model development

Stratified random sampling allocated 70% of patients to the development cohort and 30% to a held-out algorithm-comparison cohort. Missing-data handling, feature selection, hyperparameter tuning, class balancing, and model fitting used only development-cohort information. The seven fitted algorithms were then compared in the held-out cohort, and the algorithm with the highest observed AUC was designated as the final reported model. The held-out cohort did not contribute to model fitting but did contribute to algorithm selection and performance description.

Split sensitivity was assessed across 100 repeated stratified 70/30 partitions. Within each partition, the complete development workflow and all seven algorithms were rerun using only the corresponding development-cohort information. Boruta feature-selection stability was summarized across the same 100 partitions.

Within each imputed development dataset, Boruta compared observed random-forest importance with permuted shadow features and used TentativeRoughFix after a maximum of 80 runs (23). Predictors confirmed in at least three of five imputations formed the consensus set. Seven algorithms were fitted to these predictors: logistic regression, LASSO, elastic net, random forest (ranger), radial-basis-function support vector machine, Naive Bayes, and XGBoost. Hyperparameters were selected by stratified five-fold cross-validation using AUC. Minority-class upsampling for the support vector machine and Naive Bayes was confined to cross-validation training folds. Models were implemented in R using caret and algorithm-specific packages (24), and predictions were averaged across the five imputation-specific fits.

Conventional logistic regression was fitted as an unpenalized binomial model with main effects for the consensus predictors. Models were fitted separately in the five completed development datasets, and predicted probabilities were averaged for held-out comparison. LASSO and elastic net were evaluated separately as regularized models. Pairwise Spearman correlations and variance inflation factors (VIFs) were calculated for the five retained predictors in each imputed development dataset. Correlation and VIF were used to describe predictor dependence and multicollinearity, respectively, and were not interpreted as tests of the Naive Bayes conditional-independence assumption.

### 2.5 Model comparison, evaluation, and explainability

Discrimination was described in the held-out algorithm-comparison cohort using ROC curves, AUCs with DeLong 95% confidence intervals, and PR-AUC, with cohort AKI prevalence as the no-skill reference (25). Platt scaling was fitted to pooled development-set out-of-fold probabilities and applied unchanged to the held-out predictions. The operating threshold was selected by maximizing the Youden index on the Platt-calibrated development-set out-of-fold probabilities and then applied unchanged to the held-out predictions. Sensitivity, specificity, positive predictive value, and negative predictive value were calculated at this threshold. Calibration was assessed using mean predicted risk, calibration-in-the-large, calibration slope, Brier score, and a calibration curve (26). DCA compared the calibrated model with treat-all and treat-none strategies (27). In this analysis, treatment represented intensified perioperative kidney-risk management, including closer monitoring, reassessment of volume and hemodynamic status, and reduction of potentially nephrotoxic medication exposure when feasible.

Two complementary bootstrap analyses, each using 500 resamples, were performed. The model-specific bootstrap assessed the reported five-predictor Naive Bayes specification. The predictor set and algorithm were held fixed, while MICE imputation, class balancing, Naive Bayes hyperparameter optimization, and model fitting were repeated within each resample. The complete-pipeline bootstrap assessed the full data-driven development process. Within each resample, MICE imputation, Boruta feature selection, class balancing, hyperparameter optimization of the seven candidate algorithms, algorithm selection, Platt scaling, and final model fitting were repeated. Each resulting model or pipeline was evaluated in the bootstrap sample and the original cohort. For AUC, Brier score, calibration intercept, and calibration slope, optimism was estimated from the difference between bootstrap-sample and original-cohort performance, and the mean optimism was applied to the apparent full-cohort estimate. Decision-curve net benefit was not subjected to bootstrap optimism correction because it is threshold-specific and the complete development process could select different model specifications and calibrated probability distributions across resamples; DCA was therefore reported from the validation analyses.

Incremental and predictor-removal sensitivity analyses evaluated four Naive Bayes specifications, each across 100 stratified 70/30 validation partitions: the complete five-predictor model; a four-predictor model excluding uric acid; a four-predictor model excluding chloride; and a renal-function reference model containing baseline serum creatinine, preoperative BUN, and CKD or renal insufficiency. AUC, Brier score, mean predicted and observed risk, calibration intercept and slope, and DCA were summarized. DCA for these sensitivity analyses used the stored uncalibrated validation probabilities and was interpreted descriptively across the evaluated threshold range.

Sensitivity to the reference-creatinine definition was examined using the first serum creatinine measurement obtained after hospital admission and before surgery, with other KDIGO thresholds and time windows unchanged. The selected model was refitted using the alternative outcome definition and the corresponding baseline-creatinine measurement.

Model-agnostic exact Kernel SHAP values were calculated separately for each imputation-specific fit of the selected model. Twenty randomly sampled development-cohort participants served as background data, and all held-out algorithm-comparison participants were explained. SHAP values and baseline values were averaged across the imputation-specific fits and referred to the raw AKI-class probability (28). Mean absolute SHAP values summarized global importance. Beeswarm and dependence plots described prediction-level contributions, while force and waterfall plots illustrated one high-output participant.

### 2.6 Web application

An English-language R Shiny research prototype reproduced the imputation-specific fitted models, development-set scaling, pooled prediction, Platt calibration, and attribution displays. It requires complete entry of the selected predictors and flags values outside the main development-data range. Outputs include the calibrated probability, input summary, global SHAP importance, and the empirical percentile of the predicted probability in the held-out algorithm-comparison cohort.

### 2.7 Statistical analysis

Continuous variables are reported as median (interquartile range) and were compared using the Wilcoxon rank-sum test. Categorical variables are reported as count (percentage) and were compared using Pearson's chi-square or Fisher's exact test, as appropriate. Standardized mean differences (SMDs) described between-group imbalance. Analyses were performed in R version 4.6.1. *P* values were two-sided, descriptive, and unadjusted for multiple comparisons.

## 3 Results

### 3.1 Cohort and preoperative characteristics

Among 616 patients, 54 developed postoperative AKI (8.8%). The stratified 70/30 partition included 432 development patients (38 AKI events) and 184 held-out algorithm-comparison patients (16 AKI events). Median age was 83.0 years (interquartile range, 76.0–87.0), and 388 patients (63.0%) were women. Patients with AKI had longer preoperative waiting times and higher prevalences of coronary artery disease or myocardial infarction, cardiac insufficiency or heart failure, and CKD or renal insufficiency. General anesthesia was recorded in 85.2% of the cohort and did not differ significantly between groups (85.8% vs. 79.6%; *p* = 0.225). Demographic, perioperative, and comorbidity distributions are reported in Table 1.

**Table 1:** Baseline demographic characteristics, perioperative data, and comorbidities according to postoperative AKI status.

Variable	Category	Overall (*n* = 616)	Non-AKI group (*n* = 562)	AKI group (*n* = 54)	SMD	*P* value
Age, years		83.00 (76.00–87.00)	83.00 (76.00–87.00)	85.00 (80.00–89.00)	0.193	0.076
Sex	Female Male	388 (63.0%) 228 (37.0%)	356 (63.3%) 206 (36.7%)	32 (59.3%) 22 (40.7%)	0.084	0.553
BMI, kg/m^2^		20.81 (18.73–23.44)	20.81 (18.73–23.44)	20.72 (18.83–22.84)	0.080	0.980
Preoperative waiting time, days		4.00 (2.53–5.61)	4.00 (2.51–5.52)	4.46 (3.39–7.00)	0.180	0.041
Anesthesia type	General anesthesia Non-general anesthesia	525 (85.2%) 91 (14.8%)	482 (85.8%) 80 (14.2%)	43 (79.6%) 11 (20.4%)	0.163	0.225
Operative duration, min		64.00 (45.00–90.00)	64.00 (45.00–90.00)	62.50 (50.00–83.75)	0.004	0.828
Hypertension	Yes No	321 (52.1%) 295 (47.9%)	293 (52.1%) 269 (47.9%)	28 (51.9%) 26 (48.1%)	0.006	0.968
Diabetes mellitus	Yes No	116 (18.8%) 500 (81.2%)	107 (19.0%) 455 (81.0%)	9 (16.7%) 45 (83.3%)	0.062	0.670
Coronary artery disease or myocardial infarction	Yes No	81 (13.1%) 535 (86.9%)	69 (12.3%) 493 (87.7%)	12 (22.2%) 42 (77.8%)	0.266	0.039
Cerebrovascular disease	Yes No	146 (23.7%) 470 (76.3%)	129 (23.0%) 433 (77.0%)	17 (31.5%) 37 (68.5%)	0.192	0.159
Atrial fibrillation	Yes No	50 (8.1%) 566 (91.9%)	42 (7.5%) 520 (92.5%)	8 (14.8%) 46 (85.2%)	0.235	0.068
Cardiac insufficiency or heart failure	Yes No	81 (13.1%) 535 (86.9%)	67 (11.9%) 495 (88.1%)	14 (25.9%) 40 (74.1%)	0.363	0.004
Chronic kidney disease or renal insufficiency	Yes No	50 (8.1%) 566 (91.9%)	36 (6.4%) 526 (93.6%)	14 (25.9%) 40 (74.1%)	0.550	<0.001
Osteoporosis	Yes No	198 (32.1%) 418 (67.9%)	182 (32.4%) 380 (67.6%)	16 (29.6%) 38 (70.4%)	0.060	0.679
Venous thrombosis	Yes No	69 (11.2%) 547 (88.8%)	61 (10.9%) 501 (89.1%)	8 (14.8%) 46 (85.2%)	0.119	0.378
Preoperative pulmonary infection	Yes No	212 (34.4%) 404 (65.6%)	197 (35.1%) 365 (64.9%)	15 (27.8%) 39 (72.2%)	0.157	0.282
Malignancy	Yes No	52 (8.4%) 564 (91.6%)	47 (8.4%) 515 (91.6%)	5 (9.3%) 49 (90.7%)	0.032	0.797
Chronic lung disease	Yes No	86 (14.0%) 530 (86.0%)	80 (14.2%) 482 (85.8%)	6 (11.1%) 48 (88.9%)	0.094	0.527
Liver disease	Yes No	26 (4.2%) 590 (95.8%)	22 (3.9%) 540 (96.1%)	4 (7.4%) 50 (92.6%)	0.152	0.273

Values are median (interquartile range) or *n* (%), calculated from observed unimputed data using available cases. Missing values: body mass index, *n* = 230; operative duration, *n* = 361. All other Table 1 variables were complete. SMD, standardized mean difference; AKI, acute kidney injury.

All 616 patients had at least one postoperative serum creatinine measurement within 7 days after surgery, and 542 patients (88.0%) were assessed within the first 48 h. The median interval from surgery to the first postoperative creatinine measurement was 23.6 h (interquartile range, 14.7–34.0 h). Application of the serum-creatinine criteria to the available timestamped measurements identified 54 postoperative AKI events.

Among the 41 candidate predictors, 23 had at least one missing value. Missingness was concentrated in operative duration (58.6%), body mass index (37.3%), D-dimer (28.1%), and uric acid (7.8%); each remaining predictor had less than 2% missingness. The median number of missing predictors per patient was 1 (interquartile range, 1–2). Missingness in operative duration, body mass index, and D-dimer was broadly similar between outcome groups. Uric acid was missing in 13.0% of patients with AKI and 7.3% of those without AKI, and in 16.0% of patients with CKD or renal insufficiency versus 7.1% of those without this condition. Complete variable-level results are provided in Supplementary Table S1.

A sensitivity analysis using the first available serum creatinine measurement obtained after hospital admission and before surgery as the reference value identified 43 AKI events (7.0%), compared with 54 (8.8%) under the primary definition. Of the 54 patients classified as having AKI under the primary definition, 43 remained classified as AKI and 11 (20.4%) were reclassified as non-AKI. None of the 562 patients originally classified as non-AKI were reclassified as AKI. Overall agreement was 98.2%, with a Cohen's *κ* of 0.877. Under the alternative definition, the development and held-out cohorts contained 31 and 12 AKI events, respectively. The refitted five-predictor Naive Bayes model achieved a held-out AUC of 0.706 (95% CI, 0.586–0.826), compared with 0.795 in the primary analysis.

In unadjusted between-group comparisons, patients with AKI had higher baseline serum creatinine, BUN, chloride, international normalized ratio, activated partial thromboplastin time, and D-dimer. They also had lower hemoglobin, lymphocyte count, and albumin (all *p* < 0.05). Hemoglobin, BUN, and albumin had the largest laboratory SMDs. Uric acid was retained by Boruta despite no difference in the available-case comparison (*p* = 0.446; Table 2).

**Table 2:** Preoperative laboratory measures according to postoperative AKI status.

Parameter	Overall (*n* = 616)	Non-AKI group (*n* = 562)	AKI group (*n* = 54)	SMD	*P* value
Baseline serum creatinine, μmol/L	71.65 (57.38–91.60)	70.30 (56.58–90.18)	85.45 (73.97–124.45)	0.403	<0.001
Preoperative BUN, mmol/L	6.50 (5.00–8.75)	6.40 (5.00–8.50)	7.80 (6.12–11.52)	0.534	<0.001
Preoperative uric acid, μmol/L	282.00 (213.57–372.70)	280.60 (210.90–372.70)	290.30 (242.70–370.35)	0.131	0.446
Preoperative hemoglobin, g/L	100.00 (88.00–112.50)	101.00 (89.00–114.00)	91.50 (85.00–99.00)	0.626	<0.001
Preoperative neutrophil count, 10⁹/L	6.55 (4.85–8.57)	6.49 (4.88–8.56)	6.85 (4.64–8.67)	0.088	0.779
Preoperative lymphocyte count, 10⁹/L	1.05 (0.79–1.37)	1.07 (0.80–1.38)	0.87 (0.66–1.09)	0.100	0.005
Preoperative platelet count, 10⁹/L	203.00 (159.00–267.50)	202.00 (160.00–267.00)	217.50 (150.50–276.50)	0.065	0.844
Preoperative albumin, g/L	34.50 (31.10–38.30)	34.60 (31.40–38.40)	32.20 (28.40–35.70)	0.508	<0.001
Preoperative potassium, mmol/L	4.00 (3.70–4.33)	4.00 (3.70–4.32)	4.00 (3.60–4.53)	0.041	0.723
Preoperative sodium, mmol/L	137.60 (134.90–140.05)	137.40 (134.90–140.00)	138.10 (136.40–140.40)	0.256	0.132
Preoperative chloride, mmol/L	103.40 (100.20–106.10)	103.30 (100.07–106.00)	104.40 (102.00–107.30)	0.264	0.026
Preoperative calcium, mmol/L	2.12 (2.04–2.22)	2.13 (2.05–2.22)	2.09 (1.99–2.22)	0.169	0.163
Preoperative magnesium, mmol/L	0.85 (0.78–0.91)	0.85 (0.78–0.91)	0.83 (0.77–0.89)	0.261	0.222
Preoperative phosphorus, mmol/L	1.12 (0.98–1.28)	1.11 (0.98–1.27)	1.14 (0.99–1.35)	0.213	0.394
Preoperative ALT, U/L	17.00 (13.00–25.00)	17.00 (13.00–24.80)	18.50 (14.00–28.00)	0.232	0.243
Preoperative AST, U/L	25.15 (20.30–32.20)	25.10 (20.30–31.80)	27.60 (20.80–36.10)	0.246	0.283
Preoperative ALP, U/L	83.50 (66.60–105.80)	84.00 (66.68–105.65)	77.90 (66.00–120.10)	0.210	0.627
Preoperative total bilirubin, μmol/L	14.50 (10.30–19.80)	14.35 (10.30–19.78)	15.00 (10.70–20.90)	0.225	0.346
Preoperative INR	1.06 (1.01–1.12)	1.05 (1.00–1.12)	1.09 (1.04–1.14)	0.353	0.004
Preoperative APTT, s	33.85 (30.00–38.10)	33.70 (29.90–37.90)	35.50 (30.70–40.60)	0.301	0.018
Preoperative fibrinogen, g/L	3.65 (2.97–4.47)	3.64 (2.98–4.42)	4.08 (2.94–4.89)	0.218	0.157
Preoperative D-dimer, ng/mL	3790.00 (1822.50–8534.77)	3675.32 (1792.25–7547.46)	5934.00 (2666.00–13,944.00)	0.353	0.015

Values are median (interquartile range), calculated from observed unimputed data using available cases. Missing values: D-dimer, *n* = 173; uric acid, *n* = 48; INR, APTT, and fibrinogen, *n* = 12 each; ALP and phosphorus, *n* = 11 each; magnesium, *n* = 10; total bilirubin, *n* = 9; ALT and AST, *n* = 6 each; chloride and albumin, *n* = 3 each; calcium, *n* = 2; and hemoglobin, neutrophils, lymphocytes, platelets, BUN, potassium, and sodium, *n* = 1 each. SMD, standardized mean difference; BUN, blood urea nitrogen; ALT, alanine aminotransferase; AST, aspartate aminotransferase; ALP, alkaline phosphatase; INR, international normalized ratio; APTT, activated partial thromboplastin time; AKI, acute kidney injury.

### 3.2 Feature selection and algorithm comparison

Consensus Boruta selection retained baseline serum creatinine, CKD or renal insufficiency, preoperative BUN, preoperative chloride, and preoperative uric acid. Each feature was confirmed in at least three of the five imputed development datasets and was supplied to every candidate algorithm (Figure 2). Baseline creatinine, CKD or renal insufficiency, BUN, and uric acid were confirmed in all five imputed datasets, whereas chloride was confirmed in three. Across the 100 repeated partitions, baseline serum creatinine, BUN, CKD or renal insufficiency, uric acid, and chloride were selected in 100, 99, 91, 69, and 18 partitions, respectively (Supplementary Table S2). Across the five imputed development datasets, all pairwise Spearman correlations among the retained predictors were below 0.70; the largest was between baseline creatinine and BUN (mean *ρ* = 0.618). Mean VIFs ranged from 1.055 to 2.411, and the maximum VIF was 2.424 (Supplementary Table S3).

**Figure 2:**
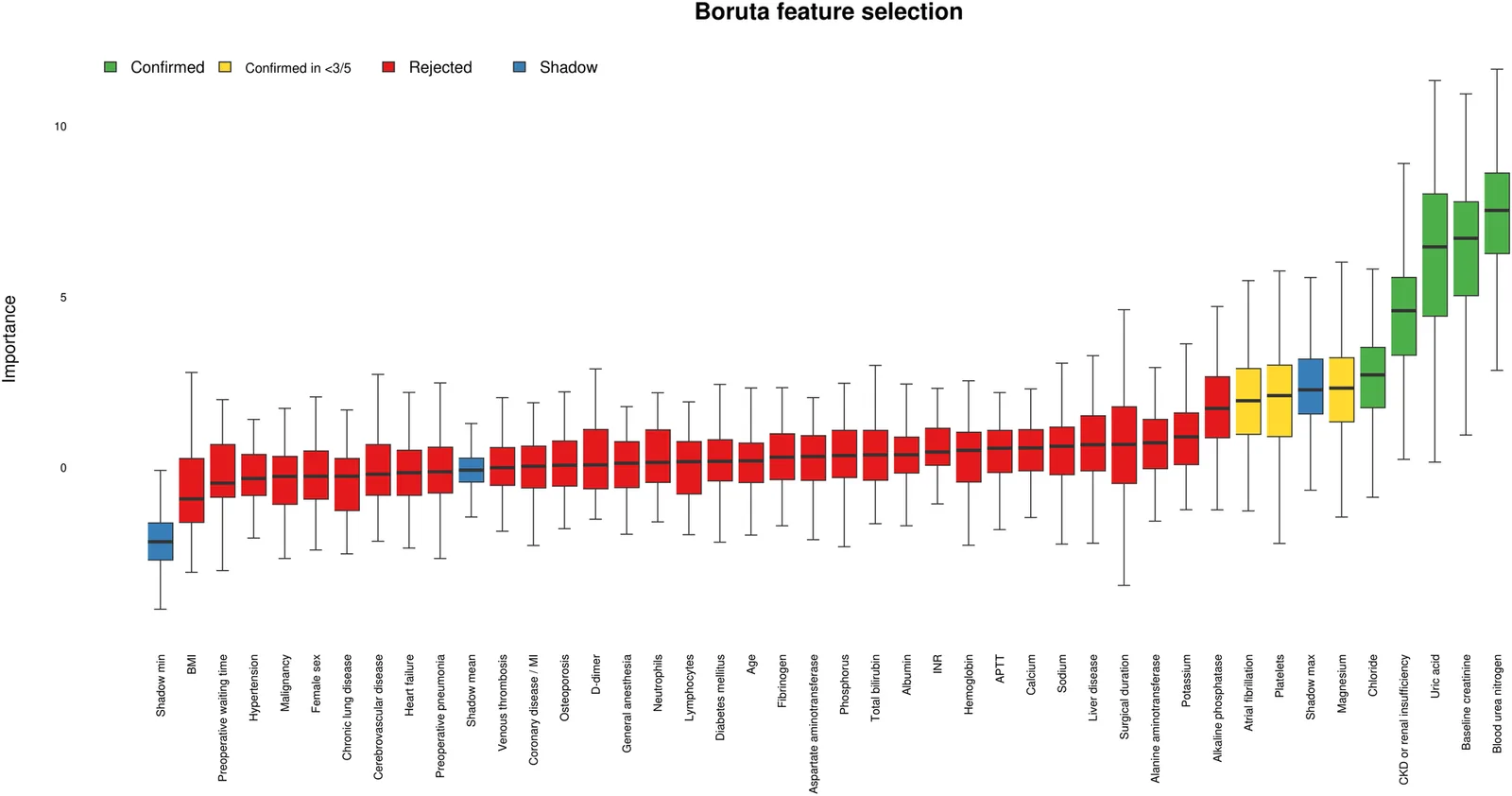
Boruta feature selection across five multiply imputed development datasets. Box plots show random-forest Z-score importance over Boruta iterations. Green indicates consensus-confirmed predictors; yellow, predictors confirmed in fewer than three imputations; red, rejected predictors; and blue, permuted shadow features.

In the held-out algorithm-comparison cohort, AUCs ranged from 0.557 to 0.795. Naive Bayes had the highest observed AUC (0.795; 95% confidence interval [CI], 0.698–0.892) and was therefore selected as the final reported algorithm. Because this cohort contributed to algorithm selection, the observed AUC is susceptible to algorithm-selection optimism. Random forest followed with an AUC of 0.778 (95% CI, 0.676–0.879), and XGBoost had an AUC of 0.743 (95% CI, 0.631–0.854). The corresponding AUCs were 0.663 for elastic net, 0.623 for LASSO, 0.589 for the radial-basis-function support vector machine, and 0.557 for logistic regression (Figures 3a,b). Supplementary Table S4 reports probability-based performance measures for all candidate algorithms.

**Figure 3:**
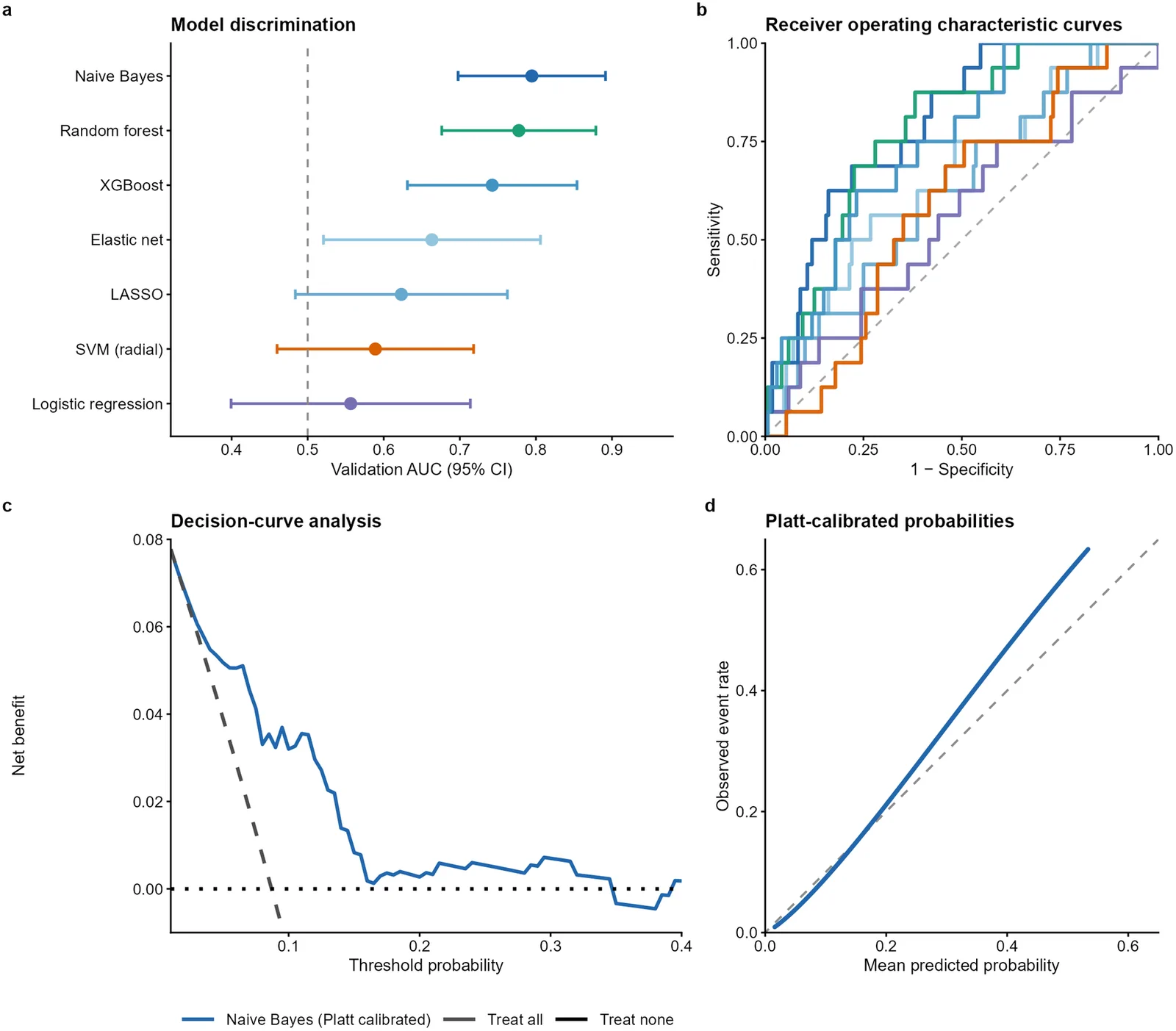
Algorithm comparison and final-model evaluation. **(a)** AUCs with DeLong 95% confidence intervals. **(b)** ROC curves for all candidate algorithms. **(c)** Decision-curve analysis for the calibrated final model. **(d)** Calibration curve for calibrated held-out predictions. AUC, area under the receiver operating characteristic curve; ROC, receiver operating characteristic.

Across 100 repeated stratified 70/30 partitions, mean validation AUCs were 0.6529 for logistic regression, 0.6776 for LASSO, 0.6857 for elastic net, 0.6880 for Ranger, 0.6251 for the radial-basis-function support vector machine, 0.7231 for Naive Bayes, and 0.6847 for XGBoost. For Naive Bayes, the median AUC was 0.7174 (IQR, 0.7041–0.7341), with a range of 0.6931–0.8158. The mean AUC of 0.7231 was used as the principal summary of internal discrimination across data partitions (Supplementary Table S5).

In the 500-repetition model-specific bootstrap, the apparent AUC was 0.9114 and mean optimism was 0.1110, yielding an optimism-corrected AUC of 0.8004 for the fixed five-predictor Naive Bayes specification. In the 500-repetition complete-pipeline bootstrap, the apparent AUC was 0.9114 and mean optimism was approximately 0.155, yielding an optimism-corrected AUC of 0.756. The complete-pipeline optimism-corrected Brier score was 0.084, the calibration intercept was 0.02, and the calibration slope was 1.70.

In the predictor-set sensitivity analyses, mean validation AUCs were 0.7231 for the complete five-predictor model, 0.7206 for the model excluding uric acid, 0.6935 for the model excluding chloride, and 0.6869 for the renal-function reference model. Corresponding mean Brier scores on the stored uncalibrated probability scale were 0.1184, 0.1170, 0.1222, and 0.1235. The complete model achieved a higher AUC than the renal-function reference model in 72 of 100 partitions. Calibration, predicted-versus-observed risk, and decision-curve summaries are provided in Supplementary Table S6.

For the final Naive Bayes model, the PR-AUC in the held-out algorithm-comparison cohort was 0.240 (95% stratified percentile bootstrap CI, 0.146–0.481), compared with a no-skill reference of 0.087. At the development-derived threshold of 0.0759 on the Platt-calibrated probability scale, there were 14 true positives, 93 true negatives, 75 false positives, and 2 false negatives. Sensitivity was 87.5%, specificity was 55.4%, positive predictive value was 15.7%, and negative predictive value was 97.9%.

Sensitivity analyses using minority-class upsampling or class weighting did not improve logistic-regression discrimination. Pooled coefficient estimates are provided in Supplementary Table S7.

### 3.3 Calibration and decision analysis

Raw Naive Bayes probabilities had a mean predicted probability of 25.9%, a Brier score of 0.118, and a calibration slope of 0.661. After Platt scaling, mean predicted risk was 9.3% and the observed event rate was 8.7%. Calibration-in-the-large was −0.076 (95% percentile bootstrap CI, −0.669 to 0.370), calibration slope was 1.225 (95% percentile bootstrap CI, 0.661–2.050), and Brier score was 0.073 (95% percentile bootstrap CI, 0.048–0.101) (Figure 3d). The AUC was unchanged. In DCA, the calibrated model had a higher estimated net benefit than treat-all and treat-none across most thresholds from approximately 0.02 to 0.345 (Figure 3c).

### 3.4 SHAP-based model interpretation

By mean absolute SHAP value, baseline serum creatinine ranked first (0.0803), followed by chloride (0.0679), BUN (0.0555), uric acid (0.0395), and CKD or renal insufficiency (0.0334). The beeswarm plot showed heterogeneous attribution patterns across the held-out algorithm-comparison cohort (Figure 4). Positive SHAP values shifted the fitted output toward higher predicted risk, whereas negative values shifted it in the opposite direction.

**Figure 4:**
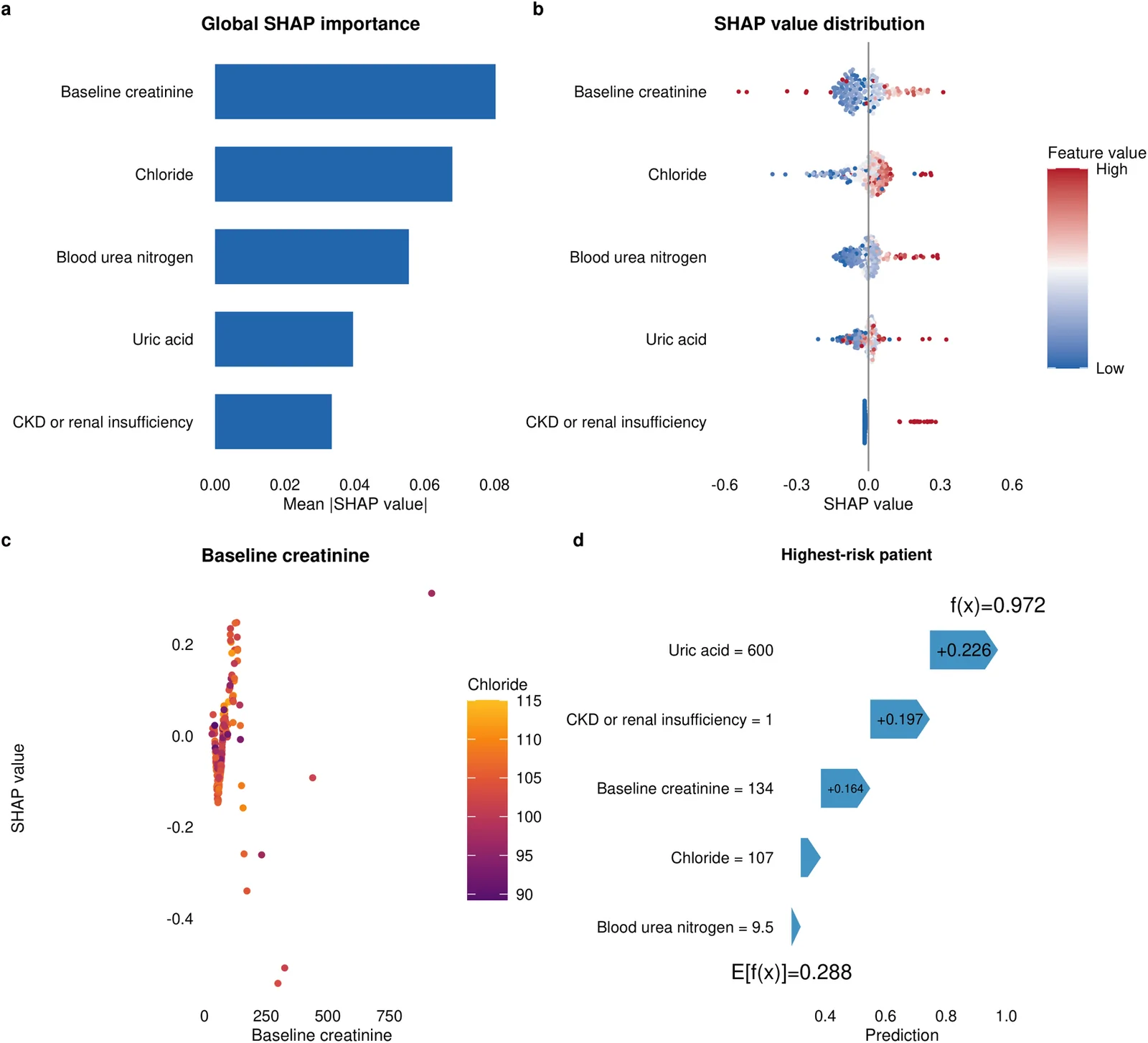
Global and local SHAP interpretation of the final Naive Bayes model in the held-out algorithm-comparison cohort. (**a**) Predictors ranked by mean absolute SHAP value. (**b**) Beeswarm plot showing prediction-level contributions; color indicates the observed feature value. (**c**) Dependence plot of baseline creatinine SHAP values, colored by chloride. (**d**) Waterfall plot for the participant with the highest raw model output. SHAP, SHapley Additive exPlanations.

Dependence plots further characterized nonlinear model-attribution patterns for all five retained predictors (Figure 5). They show how the fitted prediction changed across observed values in the multivariable feature space. Within the central range of observed chloride values, higher concentrations generally shifted the fitted output toward higher predicted AKI risk, whereas lower concentrations shifted it in the opposite direction; the pattern was less consistent at sparsely represented extremes.

**Figure 5:**
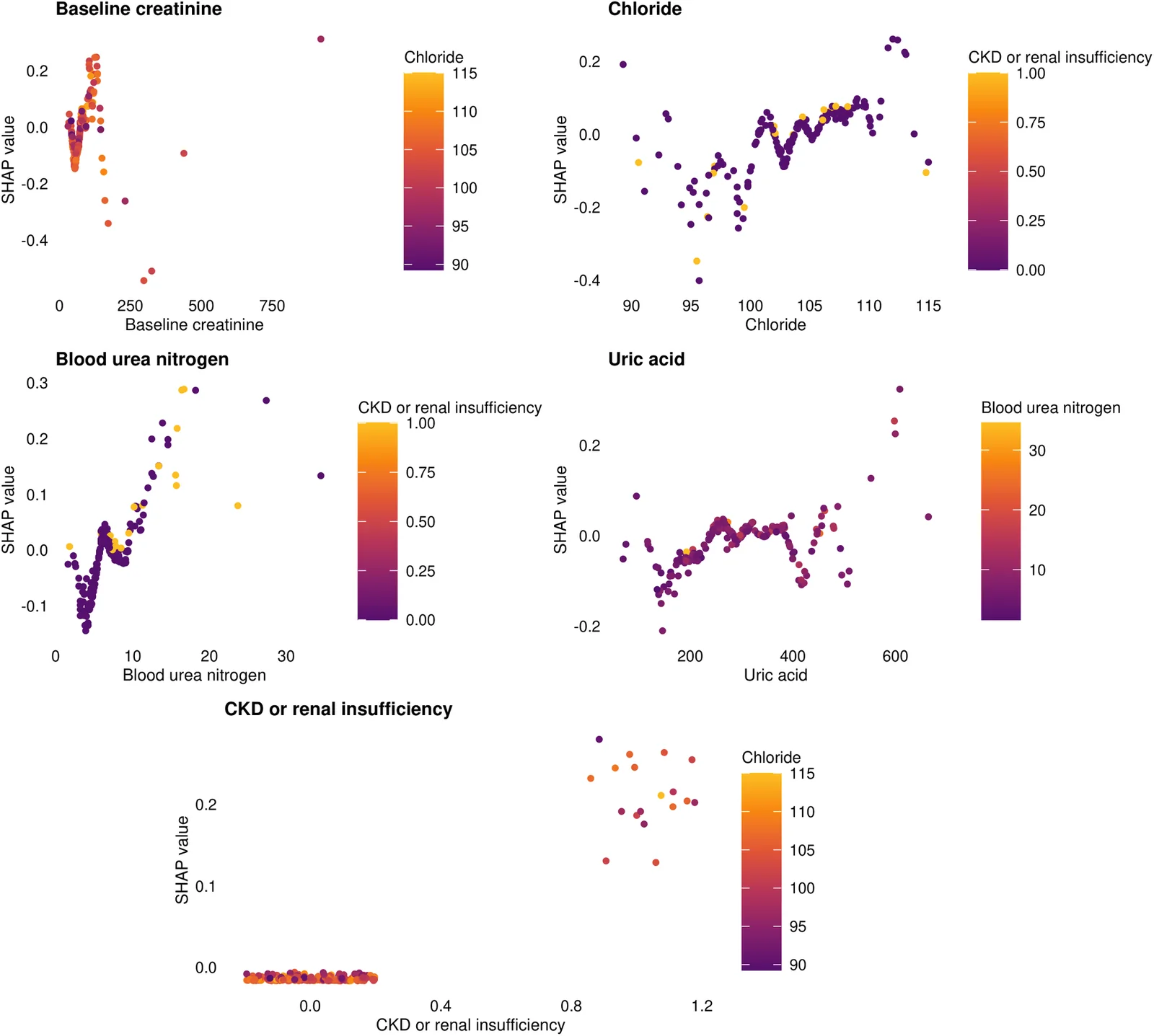
SHAP dependence plots for the five retained predictors. Each point represents one participant. The horizontal axis shows the observed feature value, the vertical axis shows its SHAP contribution to the raw model output, and color indicates the interacting feature.

The held-out algorithm-comparison participant with the highest raw model output had a raw probability of 0.972 and a Platt-calibrated probability of 0.534. The largest positive SHAP contributions came from uric acid of 599.9 μmol/L (+0.226), CKD or renal insufficiency (+0.197), and baseline serum creatinine of 133.8 μmol/L (+0.164). Chloride of 106.8 mmol/L contributed +0.0667 (Figure 6).

**Figure 6:**
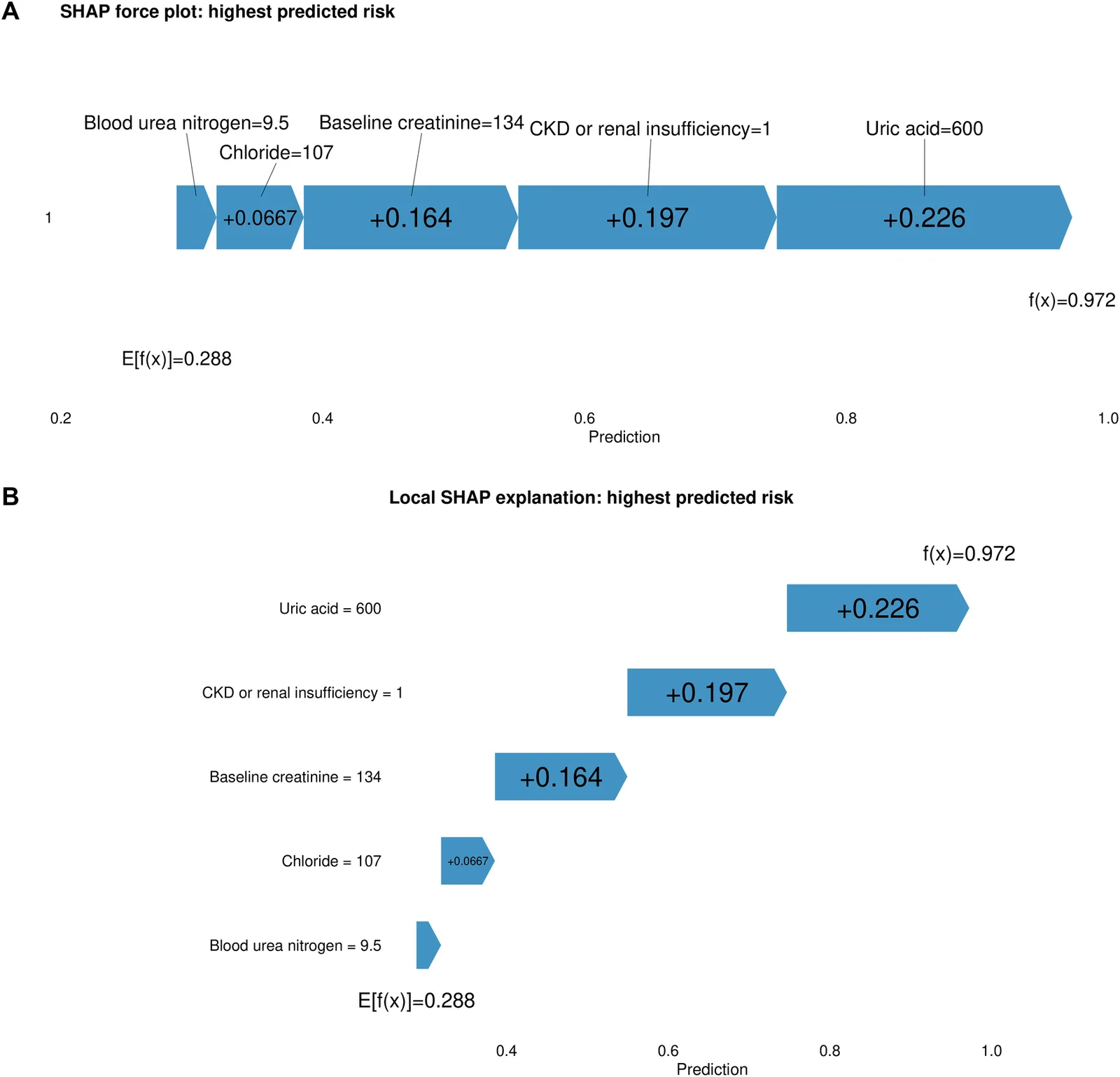
Local SHAP explanation for the participant with the highest raw model output. The force plot **(A)** and waterfall plot **(B)** show how the five predictors shift the output from the model baseline to the participant-specific estimate.

### 3.5 English-language web application

The imputation-specific models and calibration equation were incorporated into an English-language R Shiny research prototype. It displays the calibrated probability, predicted-risk percentile within the held-out algorithm-comparison cohort, entered values, global SHAP importance, and out-of-range warnings. For the example inputs in Figure 7, the estimated probability was 8.1% and corresponded to the 57th percentile among the 184 held-out algorithm-comparison participants.

**Figure 7:**
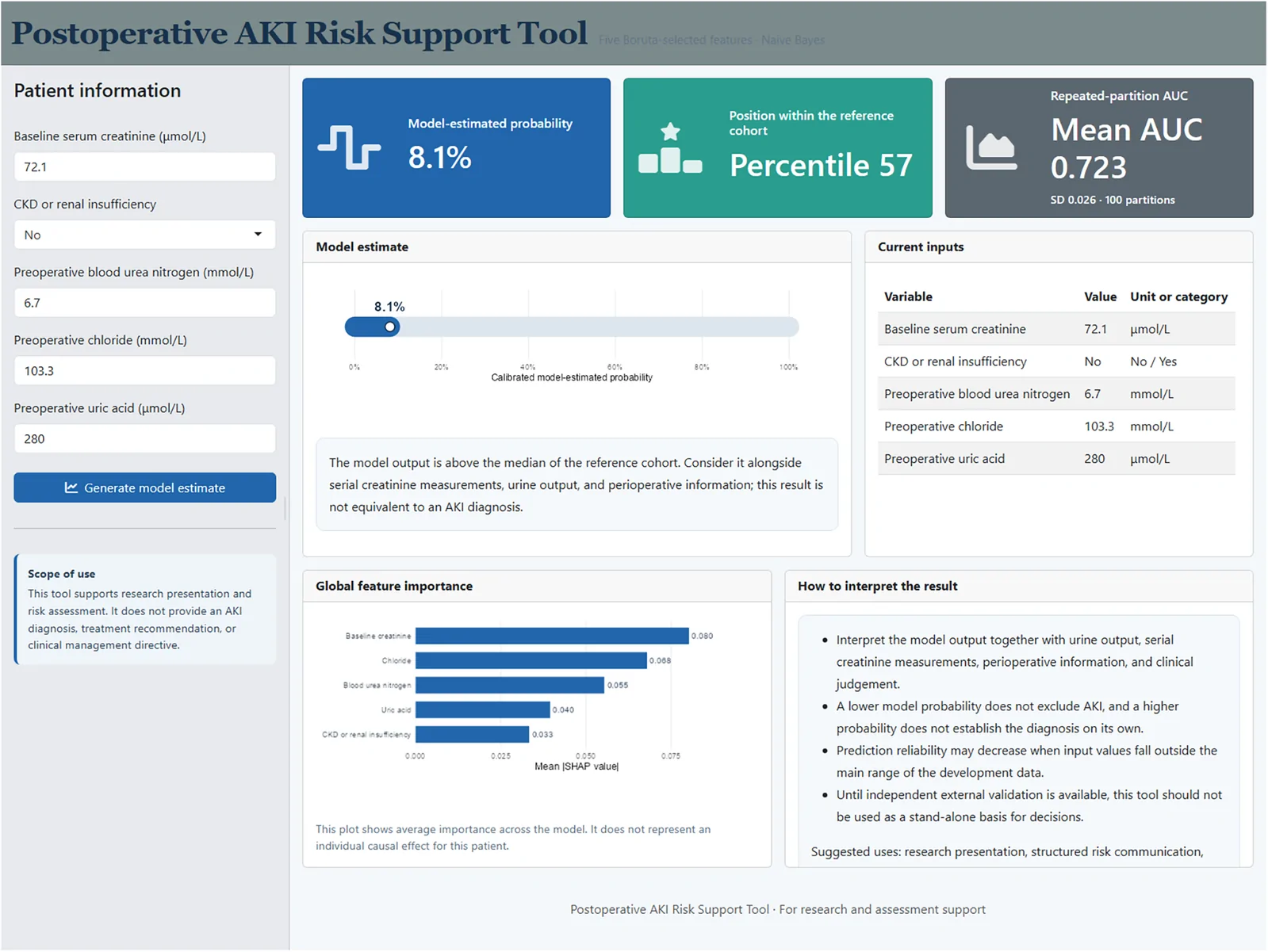
English-language R shiny research prototype. The interface displays the Platt-calibrated probability, predicted-risk percentile, entered values, global SHAP importance, and range warnings.

## 4 Discussion

This study developed an explainable preoperative model for AKI after intertrochanteric fracture surgery. Across 100 repeated stratified partitions, Naive Bayes achieved a mean validation AUC of 0.7231. Bootstrap assessment of the fixed five-predictor Naive Bayes specification produced an optimism-corrected AUC of 0.8004. When the complete development pipeline, including feature and algorithm selection, was repeated, the optimism-corrected AUC was 0.756, with a corrected Brier score of 0.084, calibration intercept of 0.02, and calibration slope of 1.70. The difference between these estimates reflects the additional selection steps included in the complete-pipeline assessment. The higher AUC of 0.795 was observed in the original algorithm-comparison cohort and is susceptible to algorithm-selection optimism. The model combined five routinely available measures of renal function and biochemical status. Platt scaling improved agreement between predicted and observed risk in the original algorithm-comparison cohort, while SHAP described predictor contributions within the fitted model.

Naive Bayes estimates class-specific predictor distributions and conventionally assumes conditional independence among predictors within outcome classes. Baseline creatinine and BUN showed moderate overall dependence (mean Spearman *ρ* = 0.618), while all VIFs were below 2.5, indicating no problematic multicollinearity. These are distinct concepts: low VIFs do not establish conditional independence. The Naive Bayes assumption was therefore regarded as approximate rather than strictly satisfied, and its observed performance does not imply general superiority over regression or other machine-learning algorithms. Minority-class upsampling and class weighting did not materially improve logistic-regression discrimination. The negative conditional coefficient for baseline creatinine is most plausibly interpreted in the context of overlapping renal-function information and instability of conditional coefficient estimates with a limited number of outcome events.

Baseline serum creatinine contributed most strongly to the model. It is a familiar marker of kidney filtration and renal reserve before surgery. Previous hip-fracture cohorts linked higher preoperative creatinine or lower estimated glomerular filtration rate with postoperative AKI (3, 7), and a meta-analysis reached a similar conclusion (4). Reduced renal reserve may limit a patient's ability to tolerate perioperative dehydration, blood loss, and hemodynamic change. Creatinine is also influenced by muscle mass and volume status, so apparently modest values may still accompany impaired kidney function in frail older adults. A multicenter perioperative study found that cystatin C provided additional prognostic information beyond creatinine-based estimates (29).

Serum chloride ranked second and showed a nonlinear model-attribution pattern. Despite its high SHAP ranking in the selected model, chloride was retained in 18 of 100 repeated partitions, indicating limited feature-selection stability across data splits. Chloride contributes to extracellular-volume and acid–base regulation, and experimental work indicates that plasma chloride can modify renal blood flow (30). Observational surgical and critical-care studies have associated hyperchloremia or rising chloride with postoperative morbidity and AKI (31, 32). Interventional evidence is less consistent, as the LICRA trial found no reduction in moderate-to-severe AKI with a chloride-restrictive strategy after cardiac surgery (33). Chloride attribution may reflect information shared with hydration, acid–base balance, renal handling, medication exposure, or unmeasured illness severity. It was therefore interpreted as a model-specific predictive marker rather than a causal risk factor or treatment target.

BUN ranked third. Higher preoperative BUN has been associated with postoperative AKI in a hip-fracture meta-analysis and appeared among candidate signals in femoral-neck fracture cohorts (4, 12). Unlike a pure filtration marker, BUN reflects renal clearance, tubular reabsorption, protein catabolism, gastrointestinal blood, nutritional state, and effective circulating volume. Its prognostic value can extend beyond creatinine in acutely ill populations (34). In the fitted model, BUN contributed information that overlapped with, but was not identical to, baseline creatinine.

Uric acid ranked fourth despite a nonsignificant available-case group comparison. Marginal group differences and multivariable model attribution describe different aspects of the data: a predictor may contribute to class-specific probability estimates without showing a marked difference in central tendency. Uric acid also shares renal and metabolic information with creatinine, BUN, and CKD or renal insufficiency. It was confirmed in all five imputed development datasets and selected in 69 of 100 repeated partitions. Studies in hospitalized and cardiac-surgery populations have linked higher uric acid with AKI (35–37), and pooled evidence supports this association across clinical settings (38). In this study, uric acid was interpreted as a model-specific predictive marker rather than a causal risk factor or treatment target.

CKD or renal insufficiency ranked fifth by mean absolute SHAP value but showed the largest baseline comorbidity imbalance. Reduced renal reserve provides plausible biological context for lower tolerance to perioperative stress, and AKI and CKD are increasingly viewed as connected syndromes (13, 39). A recorded history of CKD also adds clinical context that a single laboratory measurement cannot provide. Because several renal-function predictors were correlated, SHAP attributions may be distributed among them. The SHAP results therefore describe contributions to the fitted prediction and should not be interpreted as independent effects or causal relationships.

Precision–recall analysis provided additional context for the imbalanced outcome. At the development-derived threshold of 0.0759 on the Platt-calibrated probability scale, 89 of 184 participants were classified as higher risk, including 14 true positives and 75 false positives. The positive predictive value was 15.7%, and the operating point favored sensitivity at the cost of additional monitoring. In DCA, treatment represented closer monitoring, review of volume and hemodynamic status, and reduced nephrotoxic exposure when feasible. The estimated net benefit across most thresholds from approximately 0.02 to 0.345 represents potential clinical utility rather than an established clinical action threshold.

Across repeated partitions, the complete model had a mean AUC of 0.7231, compared with 0.7206 after excluding uric acid, 0.6935 after excluding chloride, and 0.6869 for the renal-function reference model. The small difference after removing uric acid indicates limited incremental discrimination from that variable in these data. The larger reduction after removing chloride occurred despite its limited selection stability, underscoring the distinction between importance within a fitted model and stability across data partitions. These comparisons support retaining the reported five-variable specification for the current analysis while also identifying the four-variable model without uric acid as a reasonable candidate for future external comparison. None of these results establishes a causal or treatment role for chloride or uric acid.

The R Shiny application provides an accessible implementation of the fitted preprocessing, pooled prediction, calibration, and model-attribution workflow. It requires complete input for all five predictors and flags values outside the main development-data range. The percentile output places the calibrated probability within the distribution of the 184 held-out algorithm-comparison participants. The application is a research prototype designed to support model-assisted risk review rather than replace clinical assessment, establish an AKI diagnosis, or independently direct treatment.

Key strengths were the exclusively preoperative final predictor set, feature selection across imputed datasets, comparison of seven algorithms, and evaluation beyond ROC discrimination. The cohort included 54 AKI events, making performance estimates sensitive to sampling variation (40–42). The original algorithm-comparison cohort contributed to algorithm selection, whereas the 100 repeated partitions and the two bootstrap analyses provided complementary internal assessments. The model-specific bootstrap estimated optimism conditional on the selected five-predictor Naive Bayes specification, while the complete-pipeline bootstrap additionally incorporated feature- and algorithm-selection variability. The complete-pipeline optimism-corrected AUC of 0.756 remained within the range of moderate discrimination, while the calibration slope of 1.70 indicated some compression of predicted risks. These analyses remain internal; multicenter validation and prospective evaluation should assess performance across different populations and perioperative practices.

AKI ascertainment used the serum-creatinine component of KDIGO because detailed urine-output records were unavailable. The primary baseline was the lowest observed preoperative serum creatinine concentration after admission and initial management. This choice reduced the influence of transient admission-related elevations but may have been affected by early treatment or fluid administration. When the first serum creatinine measurement obtained after admission was used instead, the number of AKI events decreased from 54 to 43, and 11 of 54 primary AKI cases (20.4%) were reclassified as non-AKI. The held-out AUC decreased from 0.795 to 0.706. These findings show that outcome classification and estimated model performance were sensitive to the reference-creatinine definition. Because postoperative creatinine surveillance followed routine clinical care, some transient, delayed, or oliguria-defined episodes may not have been captured.

Missingness was concentrated in operative duration, body mass index, and D-dimer; operative duration was not retained in the final preoperative model. MICE incorporated the available demographic, clinical, and laboratory information under a conditional missing-at-random assumption. Historical ASA classifications and preoperative medication records, including potentially nephrotoxic drug exposure, could not be reconstructed consistently after several hospital information-system changes. Future prospective data collection can standardize these variables and evaluate whether time-stamped medication, transfusion, and hemodynamic information improves a dynamically updated perioperative model.

## 5 Conclusions

This study developed and internally evaluated an explainable preoperative model for postoperative AKI after intertrochanteric fracture surgery. The mean validation AUC across repeated partitions was 0.7231. Bootstrap-corrected AUCs were 0.8004 for the fixed five-predictor Naive Bayes specification and 0.756 for the complete development pipeline, providing complementary model-specific and end-to-end estimates of internal optimism. The higher AUC observed in the original algorithm-comparison cohort should be interpreted in light of algorithm selection within that cohort. The model and accompanying R Shiny prototype are positioned as adjuncts to routine preoperative renal assessment and provide a basis for multicenter validation and prospective evaluation.

## Data Availability

The datasets presented in this article are not readily available because the datasets generated and/or analyzed during the current study are available from the corresponding author on reasonable request, subject to institutional and ethical approval. Requests to access the datasets should be directed to Hongpeng Gao, decadehp@163.com.
